# Sea Bass Primary Cultures versus RTgill-W1 Cell Line: Influence of Cell Model on the Sensitivity to Nanoparticles

**DOI:** 10.3390/nano11113136

**Published:** 2021-11-20

**Authors:** Alba Jimeno-Romero, Frederik Gwinner, Michelle Müller, Espen Mariussen, Manu Soto, Yvonne Kohl

**Affiliations:** 1 Fraunhofer Institute for Biomedical Engineering IBMT, 66280 Sulzbach, Saar, Germany; frederik.gwinner@gmail.com (F.G.); michelle.mueller@ibmt.fraunhofer.de (M.M.); yvonne.kohl@ibmt.fraunhofer.de (Y.K.); 2 Cell Biology and Environmental Toxicology Group, Research Centre for Experimental Marine Biology and Biotechnology, University of the Basque Country UPV/EHU, 48620 Plentzia, Basque Country, Spain; manu.soto@ehu.eus; 3 Norwegian Institute of Public Health, 0456 Oslo, Norway; espen.mariussen@fhi.no

**Keywords:** nanoparticles, gills, titanium dioxide, silver, polystyrene, protein corona, RTgill-W1, cell line, sea bass, primary culture, weathering, body barriers

## Abstract

Determination of acute toxicity to vertebrates in aquatic environments is mainly performed following OECD test guideline 203, requiring the use of a large number of fish and with mortality as endpoint. This test is also used to determine toxicity of nanomaterials in aquatic environments. Since a replacement method for animal testing in nanotoxicity studies is desirable, the feasibility of fish primary cultures or cell lines as a model for nanotoxicity screenings is investigated here. *Dicentrarchus labrax* primary cultures and RTgill-W1 cell line were exposed to several concentrations (0.1 to 200 ug/mL) of different nanoparticles (TiO_2_, polystyrene and silver), and cytotoxicity, metabolic activity and reactive oxygen species formation were investigated after 24 and 48 h of exposure. Protein corona as amount of protein bound, as well as the influence of surface modification (-COOH, -NH_2_), exposure media (Leibovitz’s L15 or seawater), weathering and cell type were the experimental variables included to test their influence on the results of the assays. Data from all scenarios was split based on the significance each experimental variable had in the result of the cytotoxicity tests, in an exploratory approach that allows for better understanding of the determining factors affecting toxicity. Data shows that more variables significantly influenced the outcome of toxicity tests when the primary cultures were exposed to the different nanoparticles. Toxicity tests performed in RTgill-W1 were influenced only by exposure time and nanoparticle concentration. The whole data set was integrated in a biological response index to show the overall impact of nanoparticle exposures.

## 1. Introduction

The assessment of aquatic toxicity and bioaccumulation are important components of the environmental hazard and risk assessment of all types of chemicals and are therefore included in several pieces of EU chemicals legislation. For example, with the approval of the Regulation concerning the Registration, Evaluation, Authorisation and Restriction of Chemicals [1], which also regulates nanoparticles (NPs), a great number of toxicity tests have been performed in aquatic organisms.

As a standard, tests for determining the toxicity of chemical substances in aquatic species follow OECD test guideline 203 [2], which is the most-used test for environmental risk assessment in aquatic vertebrates. This test involves the use of a large number of individuals and uses mortality as endpoint, in order to establish the concentration of the chemical that causes 50% mortality (LC_50_). According to data from the Report on the statistics on the Number of Animals used for Experimental and other Scientific Purposes the number of fish used has shown an increase, even if the total number of animals has decreased. In 2018 2,337,500 fishes were used, compared to the 1,397,462 used in 2011, and most were used in toxicological and safety evaluations (a category including 8.75% of all the experiments with animals reported), coming only second to mice [3,4]. These reports includes data on guidelines from Directive 2010/63/EU, which specifies in point 14 that mortality as a final endpoint has to be avoided when possible. Additionally, OECD tests 210 and 212 [5,6] evaluate toxicity to early-life stages and embryos of fish, respectively, and are candidates for substitution by cell-line-based assays. In this regard, several efforts showing promising results have been made to correlate the sensitivity of fish cell lines with in vivo tests that follow OECD test No. 203 [7,8,9,10,11] for better implementation of the principles of the 3Rs, following the recommendations of several regulatory frameworks (including REACH) to increase the use of alternative tests and favour this option whenever possible (replacement, reduction, refinement, proposed by Russell & Burch [12]), to the extent that an ISO guideline procedure on acute toxicity testing using a RTgill-W1 cell line assay [13] has been recently approved (not available at the time of the present research).

Most of the research on nanoparticle ecotoxicity uses either in vivo approaches or in vitro assays with invertebrate primary cultures [14,15], although fish cell lines [16,17,18] and primary cell cultures [19,20,21,22] seem to be gaining relevance because they allow the in vitro testing of an aquatic vertebrate. Furthermore, the use of established cell lines offers high reproducibility, enabling high throughput approaches that are very valuable for the screening of chemicals [11]. There are, however, some concerns regarding the loss of physiological functions, genetic drift or de-differentiation, issues that have been described in mammalian cell lines derived from tumours [23]. In the aquatic environment, if not taking into consideration diet-borne pollutants, the gill is the most exposed organ in direct contact with the environment [24,25,26]. Therefore, interactions between NPs and the different barriers such as gut and gill epithelia are of uttermost importance. Gills are crucial for ion transport activities, gas exchange, pH regulation and waste excretion. Gills have been demonstrated to be the major target organ for metal toxicity, being the dominant site of uptake for several metals [27]. As recommended by [8] for advancing the sensitivity of assays performed with fish cell-line based assays, it is best to use tissues “that reflect the specific mode of action of the chemical and to modify the culture and exposure environments to more closely mimic the in vivo exposures”. This is the rationale behind choosing the gill epithelium as the model for this work.

Additionally, we bear in mind the importance of post-manufacture modifications that pollutants undergo after being released as products. In the case of nanoparticles, they can be engineered in very different and specific ways during the manufacture process; however, once they reach the environment (biotic or abiotic), these modifications rarely remain unaffected. Among the different physicochemical changes NPs undergo in the environment, one is that biomolecules present in the media attach to NPs due to their high free surface energy. These molecules form what is known as a corona, which was thoroughly defined by [28]. The corona changes NP characteristics to such an extent that they can be considered novel nanomaterials in comparison to the stage of manufacture. Methods to investigate NP corona formation and biological relevance have been developed. Some of these methods are based on SDS-PAGE and have successfully been used in nanoparticles incubated with human plasma [29,30] and J774 macrophage cells [31]. The majority of analyses have focused on proteins due to the focus on injected nanoparticles as therapy; in any case, the presence of other biomolecules such as nucleic acids, lipids and polysaccharides is suspected and could be especially important in the water column, where complex mixtures of biomolecules coming from a great variety of species are present.

In these circumstances, it is interesting to test metallic nanoparticles (Ag and TiO_2_) along with non-metallic ones (polystyrene), as the mechanisms regulating toxicity may be different. We hope to gain deeper understanding of toxicant-handling mechanisms in the gills. While some authors argue that fish cell lines, contrary to mammalian cell lines, do not originate from tumours and thus retain their tissue-specific physiological functionalities intact, there are no established guidelines to determine the genetic stability of fish immortal cell lines [32], and it is possible that the sensitivity to pollutants is different in cells lines and primary cultures [21]. To our knowledge, there are no studies comparing the responses of fish cell lines to that of primary cultures when exposed to nanoparticles. For this purpose, we have selected the gills as model organ, due to their physiological importance, sensitivity to stress, and body-barrier role. Our research interests focus on marine environments, considered a sink for nanoparticles in the same way that they act as a sink for other pollutants [33]. Taking this into account, we selected the marine species sea bass (*Dicentrarchus labrax*) due to several factors: first, its predatory behaviour makes this species a perfect candidate for pollutant bioaccumulation, which could lead to synergic dynamics between waterborne, dietborne and accumulated pollutants. Second, it is a species with a widespread distribution in European waters and that has been studied in the field of environmental toxicology and fish physiology, providing a good background on cell morphology and pollutant turnover mechanisms, and it is of commercial relevance. As stated before, we wanted to test the capabilities of an in vitro model for toxicity screening in marine fish, and the only reference available for the same tissue was generated from a freshwater species (*Onchorynchus mykiis*). Thus, a strong factor in our choosing of *D. labrax* as marine species for this study was its ability to adapt to different salinity regimes successfully in the wild [34,35,36]. Thus, it can be used as model for saltwater, brackish waters and even freshwater, as opposed to using *O. mykiis* primary cultures, which are better suited for testing freshwater scenarios.

Starting from the primary cultures of gills from sea bass, we aim to obtain a culture of several cell types (epithelial, chloride and goblet cells) that is complex enough to give a realistic response of pollutant–gill interactions; these techniques have already been described for sea bass (Avella et al., 1994), but to date, there is no available cell line of sea bass gill epithelium. The performance and sensitivity of this mixed primary culture will be compared with that of the established (commercial) cell line from rainbow trout *O. mykiss* RTgill-W1 [37], using the endpoints cytotoxicity, metabolic activity, lysosomal membrane stability and reactive oxygen species generation as biomarkers.

## 2. Materials and Methods

### 2.1. Preparation of Nanoparticle Stock Dispersions

Nanoparticles (titanium dioxide, TiO_2_; polystyrene, PS; and silver, Ag) were synthesized by PlasmaChem GmbH (Berlin, Germany) as a liquid suspension in H_2_O, as described in [38] for TiO_2_ NPs, [39] for PS NPs and [40] for Ag NPs. Each particle was produced with two different coatings: amino (-NH_2_) and carboxyl (-COOH).

For toxicity screening experiments, all nanoparticles were dispersed in exposure media. (1) Leibovitz’s L-15 with 10% FBS (Gibco, a brand from Thermo Fisher Scientific, Darmstadt, Germany); (2) filtered seawater (from the aquaculture facility in Völklingen in which sea bass were kept, filtered to 200 µm) in serial dilutions to create working solutions of the following concentrations; 200, 40, 8, 1, 0.1 µg/mL (TiO_2_ and polystyrene NPs); 100, 10, 5, 1, 0.1 µg/mL (Ag NPs). These concentrations were selected in agreement with the OECD guideline that requires an exponential increase of the concentration to be tested and based on those used by [19]. Two sets of working solutions were prepared by sonicating stocks for 10 s and then preparing serial dilutions in the different exposure media. One set was used immediately for toxicity screening, and the other for weathering studies. Weathering conditions were aimed at investigating the adhesion of biomolecules to the NP solution, and thus solutions were incubated in falcon tubes with exposure media for 2 weeks in an agitation platform before exposure of the cells (weathered nanoparticles).

### 2.2. Characterization

The size and zeta potential of all nanoparticle sets (culture media/seawater and pristine/weathered) were characterized using a Malvern Zetasizer (Zetasizer NanoZS, Malvern Panalytical, Malvern, UK)) as described in [39]. Briefly, measurements were performed by transferring 1 mL of nanoparticle working solutions to spectrophotometer cuvettes (for size) or folded capillary cells (for zeta potential).

Electrophoretic mobility was measured by laser doppler micro-electrophoresis at 25 °C, three times with 10 to 100 sub-runs (Zetasizer NanoZS, Malvern Panalytical, Malvern, UK) and the zeta potential was calculated by Zetasizer NanoZS v 3.30 software. Results from the nanosizer were adjusted to consider refractive index and viscosity according to the methods of [41].

### 2.3. Protein Corona Quantification

These samples were processed according to [30]. Briefly, nanoparticles with an area of (1015 nm^2^) were incubated in the different exposure media (1) Leibowitz’s L15; (2) seawater) in a 1:52 ratio (area:µL incubation media). Then, nanoparticle–protein complexes were centrifuged through a sucrose cushion [42] and washed. The remaining volume was then eluted, and protein concentration as well as impurities (DNA, RNA, salts, solvents) were determined using a NanoDrop 3300 spectrophotometer (Thermo Fisher Scientific, Darmstadt, Germany). Samples were analyzed by 1D SDS-PAGE performed in a BioRad MiniProtean system using 4–20% MiniProtean Precast gels (Bio Rad. Hercules, CA, USA), followed by the Coomassie staining (Coomassie BrilliantBlue R250, Merck, Darmstadt, Germany) of the gels to determine the size of the proteins bound to the NPs.

For determining corona formation, nanoparticles were dispersed in exposure media, (1) Leibovit’z L-15 with 10% FBS and (2) filtered seawater, to create stocks with identical surface:volume ratios. Weathering conditions were aimed at investigating the adhesion of biomolecules to the NP solution, and thus solutions were incubated in falcon tubes with exposure media for 2 h and 2 weeks in an agitation platform before exposure of the cells (weathered nanoparticles).

### 2.4. Cell Cultures

#### 2.4.1. Sea Bass *Dicentrarchus labrax* Gill Primary Cells

Sea bass juveniles, prior to gonadal development, were used for gill cell isolation. Fish were purchased from an aquaculture facility in Völklingen (FRESH Corporation AG, Völklingen, Germany). Donor fishes for human consumption were maintained and euthanized according to standards for food industry by certified personnel. The gills from 10 individuals were used in the present work, and were individually factored in the dataset to explore any interindividual variations in cytotoxicity response. Data logs for water parameters (salinity, pH, temperature, nitrate and nitrite content, photoperiod, vaccination calendar) were obtained from the facility to ensure that no “seasonal” variations were introduced into the experiment.

Cells were isolated according to the protocol by [43]. Briefly, euthanized fish gills were dissected; stored in ice-cold PBS (without calcium and magnesium); supplemented with 4% pen/strep, 4% gentamycin and 4% amphotericin-B (Merck, Darmstadt, Germany); and transported on ice to the laboratory. Filaments were dissected from the gill arches, washed twice in PBS (as described before) and incubated in trypsin/PBS- at room temperature (15 min). Cell suspensions from three isolation cycles were collected in PBS with 10% fetal bovine serum (FBS, Gibco, Thermo Fischer Scientific, Darmstadt, Germany), centrifuged at 250× *g* (4 min, 18 °C) and resuspended in L15 culture media with 10% FBS, and Primocin (0.5 µL/mL) (Invivogen, San Diego, CA, USA). Primary cultures used for toxicity testing had >97% viable cells after isolation. Cells were seeded in T75 cell culture flasks in complete Leibovitz’s L15 media with L-glutamine 2 mM, 10% FBS, in an atmosphere without CO_2_ equilibration, at 18 °C. The pH (7.8) and osmolarity (355 mOsm/kg) of the culture media were adjusted according to those of sea bass serum.

#### 2.4.2. Rainbow Trout *Oncorhynchus mykiss* Gill Cell Line

The RTgill-W1 cell line was purchased from ATCC (CRL-2523 ATCC. Manassas, VA, USA) and sub-cultured in polypropylene cell culture flasks in Leibovitz’s L-15 media with 10% FBS (with L-glutamine 2 mM, 4% pen/strep; from Merck, Darmstadt, Germany) in an atmosphere without CO_2_ equilibration, at 18 ◦C. After reaching 80% confluence, cells were passaged by washing twice with PBS containing 0.48 mM EDTA ( and incubating in 0.25% trypsin for 3 min at 18 °C. Quality control, including the karyotype identity confirmation of the cell line, was carried out by the source, and provided on purchase. RTgill-W1 was kept in a laboratory designated exclusively for its culture to avoid any possible cross-contamination issues.

### 2.5. Scanning Electron Microscopy (SEM)

SEM was conducted in order to analyze the morphology, including cell surface topology, and to assess the integrity of monolayer cultures, both from sea bass primary cells and from the RTgill-W1 cell line. On passage 2, cells from both origins were seeded onto NuncTM ThermanoxTM coverslips (Thermo Fisher Scientific, Darmstadt, Germany) and cultured in 24-well plates using L-15 media. Then, cells were prepared for SEM according to [44]. Briefly, cells were washed in PBS, fixed overnight at 4 °C in 2.5% glutaraldehyde in 0.1 M sodium cacodylate buffer (Carl Roth, Karlsruhe, Germany) at pH 7.4, dehydrated in a series of ethanol (10–100%) for 30 s/step and then dried using hexamethyldisilazane (HMDS, Sigma-Aldrich, Taufkirchen, Germany) as an alternative to critical point drying. Samples were mounted onto aluminium stubs with carbon stickers and tape, coated with a thin conductive gold layer using a UNIVEX 450 B magnetron sputtering unit (Leybold, GmbH, Köln, Germany) and imaged in a field emission scanning electron microscope Phillips FESEM XL30 (Thermo Fisher Scientific, Darmstadt, Germany) at an accelerating voltage of 5 keV in secondary electron (SE)-mode.

### 2.6. Cytotoxicity Screening

Procedures for primary cells and cell line were identical. At passage 2, cells were seeded into 96-well clear polystyrene multiwell plates (Corning, Merck, Darmstadt, Germany) at a density of 5 × 104 cells/well for the screening of toxicity experiments and cultured for 24 h. Three 96-well plates per endpoint were measured (three plates, as replicates; 8 replicates per dose per NP, for each endpoint). NP exposures were performed by removing the old L-15 media, and adding 100 µL of L-15 (for RTgill-W1 and sea bass primary cells); an extra set of exposures were performed with NPs dispersed in seawater (for sea bass primary cells only) containing the concentration of the test NPs: 200, 40, 8, 1, 0.1 µg/mL (TiO_2_ and polystyrene NPs); 100, 10, 5, 1, 0.1 µg/mL (Ag NPs). To ensure the best homogeneity possible, stock solutions were sonicated in an ultrasound bath at 18 °C for 10 s prior to dilution in exposure media and the subsequent exposure of cell cultures. Weathered NPs were not sonicated to maintain the homo- and heteroaggregation processes that might have occurred during the weathering process. Wells with NPs but no cells were added as internal controls for possible interference with the endpoints measured by absorbance or fluorescence. Both cell models were exposed for 24 and 48 h, and the following endpoints were recorded: the fluorescence and absorbance intensities were corrected for background signal by subtracting the values measured in the interference control for each nanoparticle dose. Non-exposed cells were set as negative control group.

As a general rule, controls were added to check for interference of the test with nanoparticles: wells only with NPs, culture media (no cells) and the test were used. Triton (0.1%) (Roche, Merck KGaA. Darmstadt, Germany) was used as a positive control, and non-exposed cells were set as negative control unless specified otherwise. The following assays for different endpoints were selected:

WST-1 Assay (Roche, Merck KGaA. Darmstadt, Germany) was used to measure cell proliferation, cell viability and cytotoxicity. The WST-1 assay protocol is based on the cleavage of the tetrazolium salt WST-1 to formazan by cellular mitochondrial dehydrogenases. The larger the number of viable cells, the higher the activity of the mitochondrial dehydrogenases, and in turn the greater the amount of formazan dye formed. The assay was performed according to manufacture protocol and the formazan dye produced was quantified by measuring absorbance at OD = 440 nm.

Alamar Blue assay (Invitrogen, Basel, Switzerland) was used to measure cell viability. It uses the natural reducing power of living cells to convert resazurin (a nontoxic cell-permeable compound) to the fluorescent molecule resorufin. Upon entering cells, resazurin acts as an electron acceptor in the electron transport chain and is reduced to resorufin [45], which produces very bright red fluorescence that can be measured at 560/590 nm (ex/em). The assay was performed according to the manufacturer’s protocol.

The neutral red uptake assay is a cell viability assay that allows the in vitro quantification of xenobiotic-induced cytotoxicity. The assay relies on the ability of living cells to incorporate and bind neutral red, a weak cationic dye, in lysosomes. This ability is reduced when lysosome membrane integrity is compromised. Assay was performed according to the manufacturer’s protocol, and cytotoxicity was measured as absorbance at OD = 540 nm.

DCFDA—Cellular ROS Assay (ab113851. Abcam. Cambridge, UK) was used to measure cellular ROS generation. This assay uses the cell permeant reagent 2′, 7′ dichlorofluorescin diacetate (DCFDA, also known as H2DCFDA and as DCFH-DA), a fluorogenic dye that measures hydroxyl, peroxyl and other reactive oxygen species (ROS) activity within the cell. After diffusion in to the cell, DCFDA is deacetylated by cellular esterases to a non-fluorescent compound, which is later oxidized by ROS into 2′, 7′ dichlorofluorescein (DCF). DCF is a highly fluorescent compound that can be detected by spectroscopy at 495/529 nm (ex/em). Tert-butyl hydrogen peroxide (TBHP, supplied with the commercial kit) was used as positive control, and non-exposed cells were set as negative control.

### 2.7. Data Treatment

#### 2.7.1. Toxicity Assays: Endpoints and Decision Trees

For each of the two cell systems, an exploratory analysis using decision trees was carried out to determine which experimental conditions (variables) most influenced the effects each type of nanoparticle had on the measured endpoints. To this end, a conditional inference decision tree (method “ctree” from R package “party” version 1.3-3) was calculated for each cell culture system, type of nanoparticle and measured endpoint. The variables describing experimental conditions considered in the decision tree were nanoparticle coating (-COOH or -NH_2_), exposure media (L-15 or seawater), weathering of the nanoparticle (Yes or No), exposure time (24 h or 48 h), nanoparticle concentration and the measured protein concentration. The conditional inference decision tree method starts by splitting the full data set into two subgroups. This split is carried out according to the values of a variable describing the experimental conditions (e.g., split into weathered and non-weathered particles or split into data points with protein concentration < 0.012 mg/mL and protein concentration ≥ 0.012 mg/mL). A split is only carried out if it leads to two subgroups showing significantly different endpoint measurements. The two subgroups resulting from the first split are then checked for further possible splits using any of the experimental condition variables, and the algorithm stops once there are no more splits leading to subgroups with significantly different endpoint measurements.

Based on the results shown by the decision trees, the variables that significantly grouped the different treatments were selected to plot data in conventional bar graphs. Statistical analyses for these variables were performed with the aid of the SPSS.26 statistical package (SPSS Inc., Microsoft Co., Redmond, WA USA). Normality was assessed with the Shapiro–Wilk test; the homogeneity of variances was determined by Levene’s test. Multiple comparisons between experimental groups were analyzed with the non-parametric Kruskal–Wallis test followed by Dunn’s post-hoc test for multiple comparisons or two-way ANOVA, as appropriate. Significant differences were established at *p* ≤ 0.05.

#### 2.7.2. Integrative Biological Response Index

The integrative biological response (IBR) index was developed in order to integrate biochemical, genotoxicity and histochemical biomarkers [46]. In the present study, results of the different cytotoxicity endpoints were integrated into the IBR index. Hence, all outcomes from cell viability (WST, Alamar Blue), lysosomal membrane integrity (NRU) and ROS generation (DCFDA) were used to calculate the IBR index after 24 and 48 h exposure for each nanoparticle type (TiO_2_, Polystyrene, Ag) and cell model (RTgill-W1 and sea bass primary cultures). Endpoints (biomarkers) were orderly represented in the five axes of start plots. The calculation method is based on relative differences between the biomarkers in each given data set; thus, the IBR/n index (results in sup. material) is computed by summing-up triangular star plot areas (a simple multivariate graphic method) for each two neighbouring biomarkers in a given data set [46,47,48].

## 3. Results

This section may be divided by subheadings. It should provide a concise and precise description of the experimental results and their interpretation, as well as the experimental conclusions that can be drawn.

### 3.1. Nanoparticle Characterization

All nanoparticles showed an increase in hydrodynamic size after coming in contact with cell culture media L15 and seawater (Table 1). Size and Z-potential values of NPs after incubation in exposure media for 2 weeks are shown, along with the values of NPs as manufactured in dispersion media (H2O). In culture media, Ag and PS nanoparticles also shifted to larger hydrodynamic sizes, while TiO_2_ remained more stable even if it still showed a size increase up to an order of magnitude. In general, -COOH-functionalized particles showed a smaller hydrodynamic size in L15 and SW than their –NH_2_-functionalized counterparts. In the case of Ag NPs, however, -COOH-functionalized NPs showed smaller hydrodynamic size in SW but larger size in L15 than -NH_2_ coated ones.

In the review by [49], nanoparticle charge is defined as one of the main characteristics influencing uptake into mammalian cells. There, it is described that positively charged nanoparticles exhibit a higher phagocytic uptake than negatively or neutrally charged ones. Charge also determined uptake efficiency into macrophages, with charged particles (either negative or positive) showing higher uptake than uncharged ones. However, these internalization dynamics were studied without determining nanoparticle charge after coming in contact with culture media [50].

**Figure 1:**
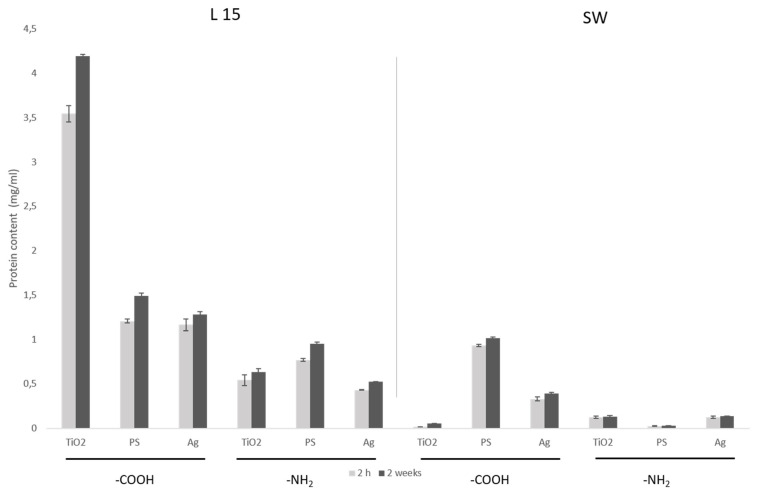
Protein content (mg/mL) measured as Abs280 from NP samples corresponding to 10^15^ nm^2^ (NP surface area) incubated in the different exposure media (L15 or sea water (SW)) for 2 h and 1 week. Size (as manufactured) of -COOH-coated NPs: (TiO_2_: 24.82 nm, PS: 61.02 nm, Ag: 89.61 nm); -NH_2_-coated NPs: (TiO_2_: 73.86 nm, PS: 141.7 nm, Ag: 93.33 nm).

Regarding protein corona formation, the main determinant was the exposure media, and particles that had been in contact with L15 showed higher amounts of protein than the ones in contact with seawater (Figure 1). Interestingly, the extent of time NPs spent in exposure media did not have a significant effect on protein content (mg/mL). Instead, the main drivers were nanoparticle type and surface modification, with -COOH-functionalized particles the ones consistently showing a higher amount of proteins bound to the nanoparticle, especially in the case of TiO_2_ NPs. It has been shown before that the surface properties of nanoparticles determine the kind of proteins that bind to them [49,51]. This can explain why, for the same surface area, -COOH-coated TiO_2_ NPs (25 nm) bound more proteins than the other, bigger particles, when incubated in culture media. In general, protein binding to -COOH-functionalized nanoparticles was size-dependent, with smaller particles binding more protein: TiO_2_ (25 nm) > PS (61 nm)–Ag (89 nm). This trend, however, did not show in—NH_2_-functionalized nanoparticles: PS (141 nm) > TiO_2_ (73 nm) > Ag (93 nm).

For nanoparticles incubated in seawater, protein binding was much lower than in L15 culture media. Protein amount was size-dependent, regardless of the coating, with the exception of the smallest (25 nm) TiO_2_ NPs, which showed a very low quantity of bound protein (Figure 1).

In the end, formation of protein corona caused a shift in the z-potential of nanoparticles, positive ones (-NH_2_) became negatively charged ones (i.e., TiO_2_: from 58.6 mV to −16.53/−9.6 mV), while negative ones (-COOH) showed values less negative than before (i.e., PS: from −60.04 mV to−12.5/−10.9 mV) (Table 1). This degree of change in z-potential was also more pronounced in NPs binding more proteins, confirming that these changes were primarily driven by the protein content of the exposure media, instead of salinity. Regardless, all NPs in exposure media (L15, SW) showed Z-potentials in the range of −16.53 mV to −4.7 mV.

It has been shown that negatively charged particles can be internalized via clathrin-mediated endocytosis [52], and thus, the modification of the overall particle charge after incubation with proteins is a determining factor for the routes of internalization and real bioavailability for the cells. In the case of NPs weathered in L15, most of the proteins are assumed to be bovine serum albumin (BSA), which is a charged protein [53] competing for NP surface.

### 3.2. Cell Culture Morphology and Characterization

**Figure 2:**
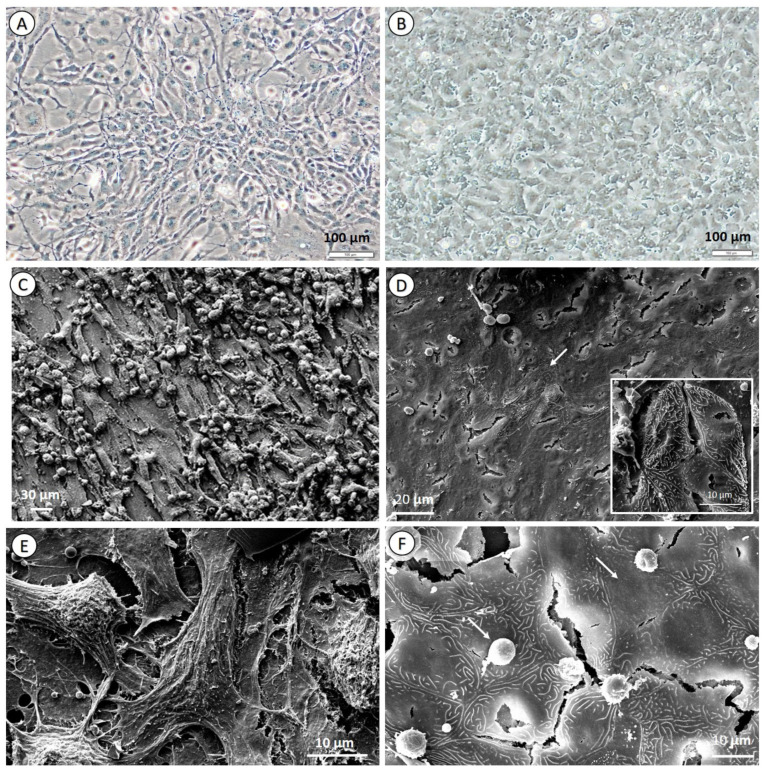
Microscopic images of RTgill-W1 cell line and sea bass primary cells. The RTgill-W1 cell line shown by light microscopy (**A**) and scanning electron microscopy (**C**,**E**), and sea bass primary cells by light microscopy (**B**) and SEM (**D**,**F**) images. Arrow in D indicates cells showing membrane microridges; detailed in the insert. Arrow in F shows cell without membrane microridges (right) and mucus deposit (left). Scale bars: 100 µm (**A**,**B**), 30 µm (**C**), 20 µm (**D**), 10 µm (**E**,**F**).

Cells corresponding to the RTgill-W1 cell line did not show any signs of polarization or surface-increasing features such as microridges and did show a fibroblast-like appearance without forming a continuous monolayer (Figure 2A). The organization of the cell cultures and the morphology of the cells contrast with those reported for the primary cultures of *O. mykiss* cells [19] but agree with the epithelial morphological description of the cell line. In the present work, the RTgill-W1 cell line seemed to be dominated by fibroblast-like cells, with a minor presence of polygonal cells; there was no sign of other cell types (Figure 2C,E). In contrast, the original paper for the generation of RTgill-W1 cell line described two types of polygonal cells: irregular ones and regular ones with distinct cell borders [37] and that cells of epithelial origin were considered the source of the cell line. The original work also reported the occasional detection of desmosomes and tight junctions, whereas they could not be detected here, at least by means of SEM, and thus in the present work, RTgill-W1 morphology resembled that described by [37]. Other authors reported that the use of serum in culture media enhances the growth of fibroblasts due to the presence of growth factors derived from platelets with mitogenic effects over this cell type [54]. Likely, this might explain the dominance of fibroblasts in RTgill-W1 cell line cultures, as the original culture was obtained using a high percentage of FBS.

Although [37] reported that their primary culture showed no defining features of specialized cells (microridges, pavement-like morphology), later works with RTgill-W1 have reported the appearance of some of those features [55] and have been successful in exposing RTgill-W1 cells to pollutants in water in the upper compartment of the cell culture inserts. However, according to our experience, it is not possible to maintain RTgill-W1 in water as exposure media, as they suffer greatly from osmotic shock, unlike sea bass primary cells. This intermittent lack of defining features could be a sign pointing out that fish cell cultures might suffer from a varying differentiation degree, loss of physiological relevance and genetic drift.

In culture, sea bass gill primary cells showed a reorganization into a continuous monolayer (Figure 2B). After the initial seeding, cells underwent division and up to four passages that spanned a total of seven weeks in culture before culture decay (Figure 2B). Cultured cells showed characteristics of different populations: flat epithelial cells; surface features without a visible membrane, probably corresponding to epithelial pavement cells; and a sub-population of cells that showed the characteristic membrane microridges of chloride cells [56]. The presence of microridges on the surface of the cell membrane is a sign of cell polarization, and it has been attributed to chloride and pavement cells (Figure 2E,F) and as a sign of their having retained the original physiologic functions, as they increase the surface area for osmotic regulation and ion exchange [57]. Chloride cells were found organized in the same fashion as in in vivo gills, as described by [58]: in the middle of a group of pavement cells, within a slightly depressed cavity (Figure 2F). A scarce amount of mucus was also present irregularly distributed on top of the cell monolayer (Figure 2F). The presence of several cell types, corresponding to the main ones found in gills, and the synthesis of mucus in the monolayer culture are strong indicators of a healthy culture that has retained its main physiological functions after being isolated from the original tissue. Especially remarkable is the presence of chloride and goblet cells, which make possible both polarization-enhanced ion exchange and the secretion of mucus, a substance known to play a vital role in the availability of nanoparticles and, consequently, their toxicity, either chemical or physical. This greater resemblance with the original tissue makes the primary culture a more suitable choice for detailed mechanistic studies, such as internalization rate determination, clearance through epithelial layers and so on. However, its short survival period in culture (4 passages spanning 7 weeks) has not completely erased the need for sea bass individuals to obtain tissue from, and thus it helps in reducing animals for experimentation, instead of completely replacing them.

### 3.3. Toxicity Assays

#### 3.3.1. Decision Trees and Individual Biomarkers

For each of the two cell systems, an exploratory analysis using decision trees was carried out to determine which experimental conditions (variables; i.e., time, exposure media, weathering, coating) most influenced the effects each type of nanoparticle had on the measured endpoints. In this analysis, individual data points are grouped into subgroups with similar endpoint measurements by repeatedly splitting the data into two subgroups. The individual splits are carried out according to the values of variables describing the experimental conditions (e.g., split into weathered and non-weathered particles), and a split is only done if it leads to subgroups showing significantly different endpoint measurements. These variables are shown in Table 2 as Factors 1, 2 and 3.

Effects exerted by NPs on sea bass cells were determined by a wider number of variables (time, exposure media, weathering, coating, protein content of the corona) whereas time, concentration and weathering were the ones determining the effects caused by NP exposure in RTgill-W1. This shows that primary cultures are sensitive to more subtle environmental factors (weathering of nanoparticles, protein content in the corona and exposure media) rather than just the pollutant-fixed variables of dose and exposure time. The biological variability of primary cultures (mixed cell types) and the wider range of conditions that affect the assay output, might make primary cultures more sensitive and suitable for in-depth mechanistic assays. If a consistent, high throughput approach were preferred, RTgill-W1 would be a more suitable model: herein, assay results were less influenced by exposure conditions and gave more consistent and reproducible results for ecotoxicological testing.

Based on the results of the culture characterization, it is evident that the different cell types present in the primary cultures play a role in these variations. For example, it has been previously reported that heterogeneous cultures (i.e., tendon) display variability in Alamar Blue readings depending on the mitochondrial abundance of the different cells [59]. The situation described there was similar to the one of the primary cultures of gill epithelium, wherein cultures have both chloride cells, which are rich in mitochondria, and pavement cells, which are less metabolically active. Accordingly, RTgill-W1 was dominated by a single cell type (Figure 2), making the results more reproducible, because the proportion of cell types did not vary from culture to culture and were not affected by physiological fitness of the individual from whom the cells were obtained.

After the separation of data based on the significant variables revealed by the decision trees, significant differences between subgroups of means were analyzed for each nanoparticle concentration and in vitro model. In the following sections, only data from endpoints that showed significant differences—based on the clustering obtained from decision trees—are displayed.

#### 3.3.2. Exposure to TiO_2_ Nanoparticles

**Figure 3:**
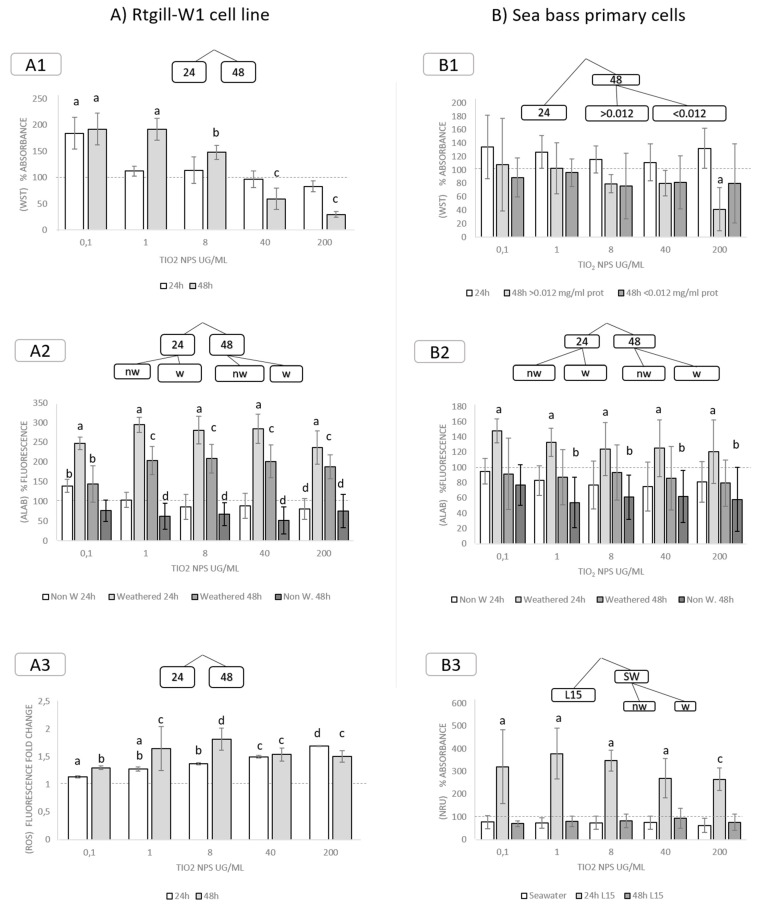
Significant effects for each measured endpoint after splitting data based on decision tress. Effects of exposure to TiO_2_ NPs in (**A**) RTgill-W1 cell line measured as cell viability (A1, WST; A2, Alamar Blue), and ROS generation (A3, DCFDA); and (**B**) Sea bass primary cells measured as cell viability (B1, WST; B2, Alamar Blue), and lysosomal membrane stability (B3, NRU). Data has been fragmented based on decision tree results. Letters indicate groups of the same significance (a, b, c, d) and significant intra- and inter-group differences. (*p* < 0.05) Bars indicate standards deviation. 24: 24 h exposure, 48: 48 h exposure, nw: non-weathered, w: weathered, SW: sea water.

In RTgill-W1, cell viability (as measured as WST-1 absorbance) decreased significantly after 48 h of exposure to 40 and 200 µg/mL of TiO_2_ nanoparticles. With lower doses (0,1; 1 and 8 µg/mL), results showed a significant increase in the signal, which can be related to increased metabolic activity (Figure 3A1). In cells exposed to weathered nanoparticles, cell viability (metabolic activity) measured as Alamar Blue absorbance showed a significant increase, regardless of exposure time (Figure 3A2). ROS generation increased in all exposure doses, in a dose-related manner (Figure 3A3). Taking into consideration that none of the other parameters varied, this response could be due to an increased metabolism because of sublethal stress. No significant changes in neutral red uptake were measured (data not shown).

In sea bass primary cultures, exposure to TiO_2_ NPs at a concentration of 200 µg/mL for 48 h caused a significant decrease in viability (measured as WST-1 absorbance) in conditions wherein nanoparticles showed bound protein concentrations > 0.012 mg/mL. Otherwise, no significant differences were detected (Figure 3B1). When metabolic activity was measured via Alamar Blue, the main factors determining effects were weathering and time. Exposure to weathered nanoparticles resulted in an increase in cell metabolism after 24 h (compared to controls and non-weathered nanoparticles). Non-weathered nanoparticles followed a predictable time-dependent effect, wherein metabolism was lower after 24 h and significantly lower after 48 h of exposure (Figure 3B2). Neutral red uptake was significantly higher in cells exposed for 24 h in culture media (L15), compared to those exposed in SW or for 48 h in L15 (Figure 3B3). ROS generation showed no significant differences with controls, in any case (data not shown).

#### 3.3.3. Exposure to Polystyrene (PS) Nanoparticles

**Figure 4:**
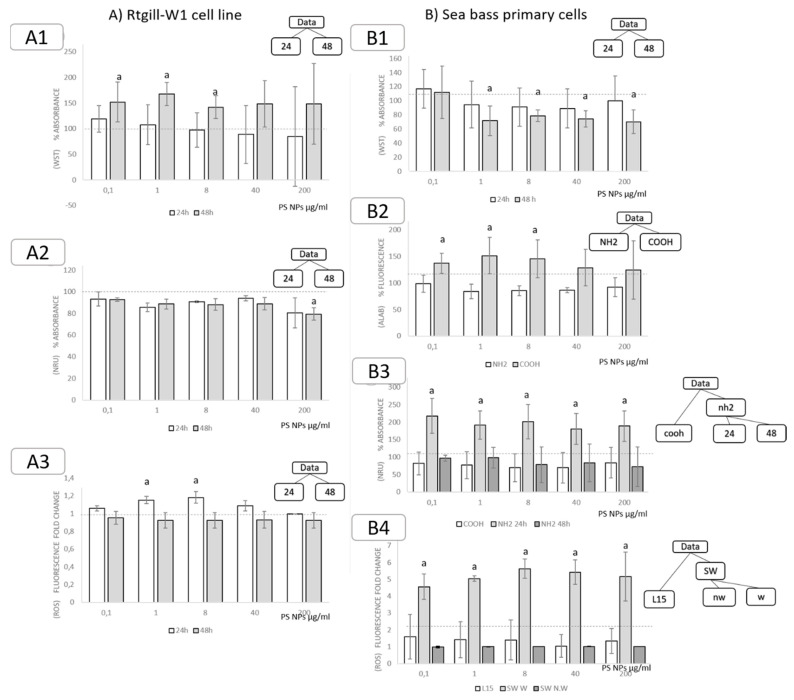
Significant effects for each measured endpoint after splitting data based on decision tress. Effects of exposure to PS NPs in (**A**) RTgill-W1 cell line measured as cell viability ( A**1**, WST), lysosomal membrane stability (A2, NRU) and ROS generation (A3, DCFDA); and (**B**) Sea bass primary cells measured as cell viability (A1, WST; A2, Alamar Blue), lysosomal membrane stability (A3, NRU) and ROS generation (A4, DCFDA). Data has been fragmented based on decision tree results. Letters indicate significance groups (*p* < 0.05). 24: 24 h exposure, 48: 48 h exposure, nw: non-weathered, w: weathered, SW: sea water. L15: cell culture media L15. NH_2_: NH_2_-surface functionalization, COOH: COOH-surface functionalization.

The RTgill-W1 cell line showed no significant mortality, and a significant increase in metabolic activity (measured as WST-1) was recorded in cells exposed for 48 h to 0.1, 1 and 8 µmg/mL PS nanoparticles (Figure 4A1). Lysosomal membrane stability (measured as NRU) significantly decreased in cells exposed for 48 h to 200 µg/mL PS NPs (Figure 4A2). ROS production significantly increased after 24 h of exposure to 1 and 8 µg/mL (Figure 4A3).

A significant decrease in cell viability (measured as WST-1) was detected in sea bass cells exposed to 1, 8, 40 and 200 µg/mL PS NPs for 48 h (Figure 4B1). Additionally, an increase in metabolism (measured as Alamar Blue) was observed in sea bass cells exposed to 1, 8 and 40 µg/mL -COOH-functionalized particles, regardless of exposure conditions (Figure 4B2). Regarding NRU, Exposure to NH_2_-functionalized PS NPs resulted in an increase in the dye uptake (Figure 4B3), probably related to the increase in metabolic activity detected before. Exposure to weathered PS NPs in seawater caused a significant increase in ROS production in all the doses, compared to exposure to PS nanoparticles in L15 media (both weathered and non-weathered) and in seawater (non-weathered) (Figure 4B4).

#### 3.3.4. Exposure to Ag Nanoparticles

**Figure 5:**
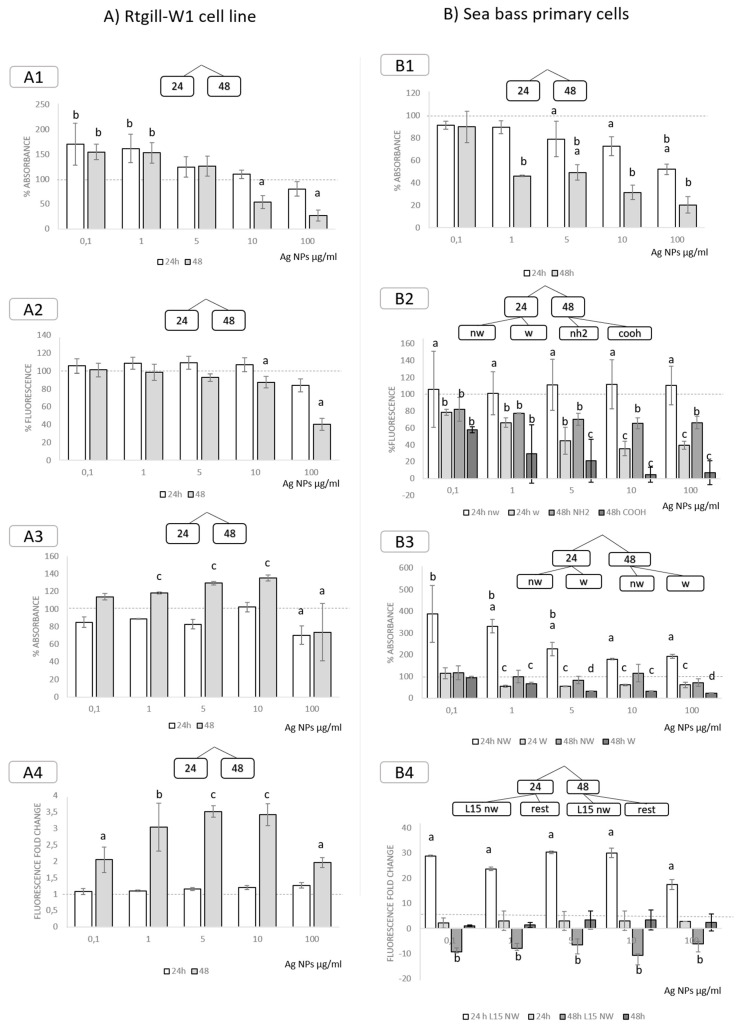
Significant effects for each measured endpoint after splitting data based on decision tress. Effects of exposure to Ag NPs in (**A**) RTgill-W1 cell line measured as cell viability (A1, WST; A2, Alamar Blue), lysosomal membrane stability (A3, NRU) and ROS generation (A4, DCFH-DA); and (**B**) sea bass primary cells measured as cell viability (A1, WST; A2, Alamar Blue), lysosomal membrane stability (A3, NRU) and ROS generation (A4, DCFH-DA). Data has been fragmented based on decision tree results. Letters indicate significant intra- and inter-group differences according to Duncan post-hoc test (*p* < 0.05). 24: 24 h exposure, 48: 48 h exposure, nw: non-weathered, w: weathered, SW: sea water. L15: cell culture media L15. NH_2_: NH_2_-surface functionalization, COOH: COOH-surface functionalization.

Regarding the RTgill-W1, WST significantly increased after exposure to 0.1 and 1 µg/mL in both 24 and 48 h exposure times. Exposure to 10 and 100 µg/mL, however, caused a significant decrease of cell viability after 48 h of exposure (Figure 5A1). A significant decrease in cell viability after 48 h of exposure to 10 and 100 µg/mL was also detected by Alamar Blue measurements. (Figure 5A2). A significant increase in NRU was observed after 48 h of exposure to all the doses, except for exposure to 100 µg/mL, which resulted in a significant decrease both after 24 and 48 h (Figure 5A3). Exposure to Ag NPs significantly increased ROS production after 48 h, this increase being significantly higher in cells exposed to 5 and 10 µg/mL AG NPs (Figure 5A4). All of these results indicate that there was an acute response wherein RTgill-W1 cells increased metabolic activity as means of coping with stress, but after 48 h, these mechanisms were insufficient, resulting in an increase in ROS (as antioxidant activity was reduced) and accompanying cell death. It has been reported that exposure to sublethal doses of metals result in increased antioxidant activity measured as GSH and GST [60].

The exposure of sea bass cell cultures to Ag NPs resulted in significant effects for all the biomarkers measured (Figure 5B). Both markers of cell viability and metabolism were significantly decreased by Ag NPs exposure. Cell viability (measured as WST) showed a significant decrease after exposure to 8, 40 and 200 µg/mL for 24 h and from 1 µg/mL and all higher doses after 48 h (Figure 5B1). Metabolic activity (measured as Alamar Blue) was significantly decreased in cells exposed to weathered NPs for 24 h and to weathered and non-weathered particles after 48 h. The only exception was exposure to non-weathered Ag NPs for 24 h, a condition which significantly increased metabolism (Figure 5B2). This could be related to the increased uptake activity/lysosomal membrane stability measured by NRU, wherein exposure to non-weathered Ag NPs for 24 h to all doses and for 48 h to 40 and 200 µg/mL caused a significant increase in the uptake of the compound (Figure 5B3). Among the rest of the conditions, exposure to -COOH-functionalized NPs for 48 h was the one causing a more severe metabolic impairment, in a dose-dependent way, with—NH_2_-coated particles having a lesser effect. Cells exposed to non-weathered Ag NPs in culture media (L15) showed a significant increase in ROS production, for all doses. After 48 h, this same treatment produced ROS value readings that were significantly below controls levels, as a result of cell mortality. The rest of the exposure conditions did not cause any significant difference in ROS production when compared to controls (Figure 5B4).

As expected, Ag nanoparticles had the most severe effects for all the endpoints measured, when compared to TiO_2_ and PS ones, which are deemed non-toxic. Ag+ causes high toxicity to aquatic organisms, acting as a disruptor of ion regulation in gills where it inhibits sodium uptake [61]; part of the toxicity observed here could be a result of dissolved silver from Ag NPs, although additional mechanisms take place. For example, fish (juvenile *Cyprinus carpio*) gills are affected by exposure to Ag NPs in vivo, showing signs of necrosis, aneurism, shortening of secondary lamellae, collapse of secondary lamella and other deleterious conditions [62], among them aneurism and oedema, the conditions associated with ion imbalance. In seawater, Ag ions compete with other cations for the binding sites [61], and it has been hypothesized that, in seawater, the toxicity of NPs in general and dissolved silver in particular would be less than in other exposure media. In the present work, however, the variation in exposure media (L15 or SW) did not result in a significant split in the data set—according to the exploratory analysis (Table 2)—for sea bass primary cells. Rather, it was the weathering state of the NPs that influenced the outcome of the exposures.

In some cases, exposure to TiO_2_ and PS NPs also resulted in increased levels of metabolic activity and cell viability (measured as Alamar Blue and WST) in both sea bass primary cells and RTgill-W1 cell line. It has been discussed previously that this could be caused by an increase in metabolic activity due to an increase in antioxidant response and general MXR activation. Accordingly, Ref. [63] reported an increase in GST and EROD activity in juvenile *Pomatoschistus microps* exposed to 0.2 mg/L Au NPs. In addition, Ref. [64] reported that the exposure of the RTgill-W1 cell line to 7 µg/mL tungsten carbide NPs in L15 also resulted in an increase of the metabolic activity (measured as Alamar Blue). This increase in metabolic activity has been already described as part of the processes aimed to cope with pollutant insult [65]. However, another possible explanation is related with the increased distance between the polluted outer media and the bloodstream after the exposure of fish to metals (Cr, Cd, Cu, Zn, Ag NPs) in vivo as a result of gill hyperplasia, a phenomenon occurring when gill cells divide in response to stress [27,66,67,68].

#### 3.3.5. Integrated Biological Response (IBR) Index

**Figure 6:**
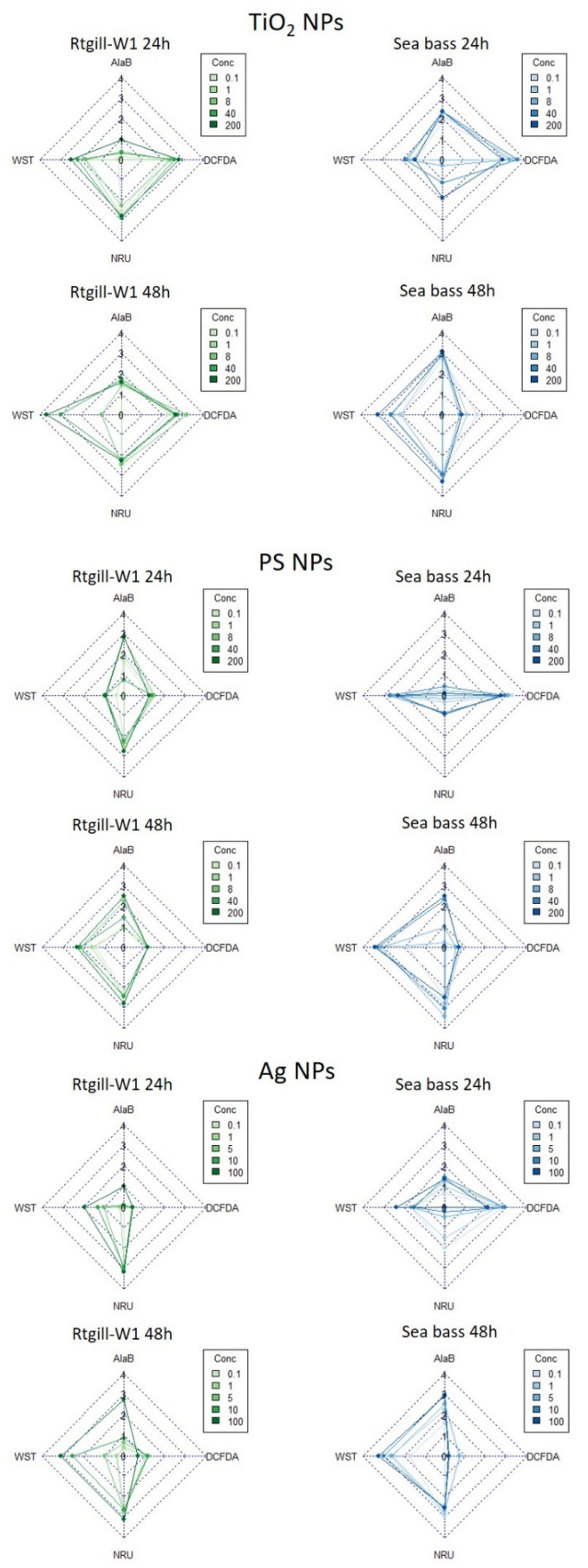
Radar plots for S-values corresponding to exposures toTiO_2_, PS and Ag nanoparticles. Legends show exposure concentrations, the Controls being the ones corresponding to a value of 0—no effect—in the radar plot.

In order to give an overview of the overall effects of nanoparticle exposure, an integrated biological response (IBR) analysis was carried out (see Methods section for details). The radar plots in Figure 6 show the relevance of the individual biological responses of the two cell culture systems after exposure to the individual nanoparticles for 24 and 48 h. After exposure to TiO_2_ NPs (Figure 6), ROS generation was the main observed response in sea bass cultures after 24 h. In RTgill-W1 ones, there was a greater degree of response both in ROS generation and NRU. After 48 h, however, the effects observed in sea bass cultures shifted towards cytotoxicity, increasing the contribution of WST-1, Alamar Blue and NRU measurements while decreasing the relevance of ROS generation. This was also observed in RTgill-W1, although the shift in this case was more pronounced towards the contribution of WST. Exposure to PS NPs (Figure 6) resulted in a greater effect in cell viability to RTgill-W1 measured as Alamar Blue and NRU than the rest of the parameters, whereas after 48 h the weight shifted towards cell viability measured as WST-1. In sea bass primary cultures, exposure to polystyrene NPs resulted in greater ROS generation and decreased cell viability as measured by WST. After 48 h, all of the cell integrity markers showed a similar weight (WST, Alamar Blue, NRU), and the effect of ROS generation was minimal. (Figure 6) Exposure to Ag NPs (Figure 6) for 24 h resulted in generally low ROS generation in RTgill-W1 cells, and a strong effect in NRU in all of the concentrations tested. In sea bass cells, however, ROS generation was the main contributor to the effects observed, followed by a slight influence of cytotoxicity detected by WST-1. After 48 h, exposure to Ag nanoparticles showed a similar profile for both RTgill-W1 and sea bass cultures, with a very low effect of ROS, and an increase of the effect measured as cytotoxicity (WST-1 and Alamar Blue) and lysosomal membrane integrity (NRU).

Thus, the responses observed in the different biomarkers were influenced by different variables depending on the cell type cultured. Sea bass primary cells showed a wider range of variability, and clustering of data showed that those results depended on several intrinsic characteristics of nanoparticles (coating) and other extrinsic ones such as weathering. Conversely, the RTgill-W1 cell line showed less variability in the results, which were determined mostly by exposure time (24 or 48 h) and NP concentration. Exploratory analysis helped to identify the variables influencing toxicity to gill cells, and thus proved to be a useful tool to use when trying to identify key drivers of toxicity among nanoparticle and environmental characteristics, which will help in the development of safe-by-design nanomaterials and the ontological grouping of materials.

## 4. Conclusions

In light of the results obtained in the present work, the most cost-effective and reproducible approach for high throughput screening was the RTgill-W1 cell-line; however the most sensitive and realistic setup was based on the primary cultures of sea bass because of the heterogeneous cell-type composition; and therefore, the selection of the system depends on the objective of the work. For instance, for a biomonitoring screening the use of cell lines is more accurate, whilst mechanistic studies deserve more a complex approach based on primary cultures. It is worth mentioning that further research into the optimization of culture conditions for sea bass gill primary culture might extend their life period, as the adjustments described in this work such as adjusting the osmolarity to match that of the serum of the animals from where the cells were harvested, adding L-glutamine to the culture media and an increase in FBS (from 5% to 10%), have already improved it greatly, and thus make them more suitable for routine toxicity screenings when gill cultures from a fish with a wide salinity tolerance range are needed.

## Figures and Tables

**Table 1:** Characterization of NP as manufactured in H_2_O and dispersed in exposure media (L15, Leibovitz’s culture medium; SW: Seawater).

Material	Coating	Media	Z-Ave (nm)	St.Dev	Z-Pot (mV)	St.Dev
TiO_2_						
	NH_2_	H_2_O	73.86	1.78	58.6	5.41
	NH_2_	L15	137.33	10.21	−16.53	2.35
	NH_2_	SW	1567.3	60.8	−11.9	1.529
	COOH	H_2_O	24.82	5.37	−29.2	3.62
	COOH	L15	178.7	16.3	−14.9	1.627
	COOH	SW	2103.3	45.6	−9.6	1.816
Polystyrene						
	NH_2_	H_2_O	141.7	2.04	54.6	1.92
	NH_2_	L15	918.0	61.6	−9.3	1.519
	NH_2_	SW	5542.5	928.6	−8.1	1.627
	COOH	H_2_O	61.02	1.3	−60.04	0.987
	COOH	L15	7640.7	609.6	−12.5	2.003
	COOH	SW	9643.3	1682.1	−10.9	0.123
Ag						
	NH_2_	H_2_O	93.33	2.04	18.4	0.1
	NH_2_	L15	5561.3	1576.1	−13.9	0.354
	NH_2_	SW	1.90 × 10^7^	5.98^5^	−12	0.168
	COOH	H_2_O	89.61	0.285	−61.1	1.4
	COOH	L15	7.15 × 10^6^	6.18 × 10^6^	−5.6	1.106
	COOH	SW	8417.0	666.3	−4.7	0.026

**Table 2:** Results of the exploratory analysis determining the weight of each variable in the results of the biomarker tests. Factors 1, 2 and 3 correspond to the significant splits in data detected after the exploratory analysis. The name of the corresponding variable for each endpoint is given along with the significance of the split.

Scheme 1.							RTgill W1				
TiO_2_							TiO_2_				
Assay	Factor 1	Sig f1	Factor 2	Sig f2	Factor 3	Sig f3	Assay	Factor 1	Sig f1	Factor 2	Sig f2
**ROS**	Time	0.003	Coating (48 h)	0.046			**ROS**				
**NRU**	ExpMedia	<0.001	Time (L15)	<0.001	Weathering (48 h)	0.011	**NRU**	Time	0.027		
**WST**	Time	0.001	Protein (48 h)	0.01			**WST**	Concentration	0.004		
**AlaB**	Weathering	<0.001	Time (Y)	0.003	ExpMedia (48 h)	0.008	**AlaB**	Weathering	<0.001	Coating (Y)	0.032
**PS**							**PS**				
**Assay**	**Factor 1**	**Sig f1**	**Factor 2**	**Sig f2**	**Factor 3**	**Sig f3**	**Assay**	**Factor 1**	**Sig f1**	**Factor 2**	**Sig f2**
**ROS**	ExpMedia	<0.001	Weathering (SW)	<0.001			**ROS**	Time	<0.001		
			Time (L15)	<0.001							
**NRU**	Coating	0.007	ExpMedia (a)	0.01	Time (a, L15)	0.023	**NRU**	Conc	0.003		
**WST**	n.a						**WST**	Weathering	0.012		
**AlaB**	Coating	<0.001					**AlaB**				
**Ag**							**Ag**				
**Assay**	**Factor 1**	**Sig f1**	**Factor 2**	**Sig f2**	**Factor 3**	**Sig f3**	**Assay**	**Factor 1**	**Sig f1**	**Factor 2**	**Sig f2**
**ROS**	Time	0.001	Weathering/ ExpMedia	0.044/0.009	ExpMedia/Weathering	<0.001/0.015)	**ROS**	Time	<0.001	Conc	0.032
**NRU**	Weathering	<0.001	Time (non w)	0.011	ExpMedia (non w)		**NRU**	Time	<0.001		
**WST**	Time	0.001					**WST**	Conc	0.001		
**AlaB**	Time	0.002	Weathering (24 h)	<0.001			**AlaB**	Conc	0.002	Time (conc<10)	0.002
			Coating (48 h)	<0.001							
